# MS-Based Crevicular Fluid Proteomics for the Study of Periodontal and Peri-Implant Conditions: A Systematic Review

**DOI:** 10.1021/acs.jproteome.5c01217

**Published:** 2026-06-24

**Authors:** Laís de Paula Sumback Sivila Souza, Débora Reis Dias, Giuliano Portolese Sversutti Cesar, Débora de Almeida Bianco, Mauricio Guimarães Araújo, Flavia Matarazzo

**Affiliations:** † Department of Dentistry, State University of Maringá−CEP, 87013-010 Maringá, Brazil; ‡ Department of Dentistry, State University of Maringá−CEP, 87013-010 Maringá, Brazil; § Department of Dentistry, State University of Maringá−CEP, 87013-010 Maringá Brazil; ∥ Department of Dentistry, State University of Maringá−CEP, 87013-010 Maringá Brazil; ⊥ Department of Dentistry, State University of Maringá−CEP, 87013-010 Maringá, Brazil; # Department of Dentistry, State University of Maringá−CEP, 87013-010 Maringá, Brazil

**Keywords:** biomarkers, crevicular fluid, liquid chromatography, mass spectrometry, periodontitis, peri-implantitis

## Abstract

This systematic review aimed to evaluate the current literature on mass spectrometry (MS)-based proteomic analysis of the crevicular fluid in different periodontal and peri-implant conditions, and to summarize methodological differences among studies. A search of electronic databases was conducted and clinical studies using MS-based proteomics in gingival (GCF) and/or peri-implant crevicular fluid (PICF) were considered for inclusion. The findings were synthesized and methodological variations described. A modified QUADOMICS tool was applied for risk of bias assessment. Thirteen studies; five longitudinal and eight cross-sectional were analyzed. Patients ranged from 10 to 190, with 42 to 3070 human proteins identified. Sample preparation and preanalytical procedures differed among studies. Protein identification, characterization and quantification were conducted using different algorithms and computer software against different databases. Different strategies were used to select distinctive proteins. Six studies attempted at biomarker development using different protein selection and validation criteria. While six studies presented moderate quality, seven were considered to be low quality. The present findings emphasize the need for methodological harmonization, including standardized protocols for GCF/PICF collection, harmonized proteomic workflows, multicenter longitudinal validation studies, and targeted mass spectrometry approaches for biomarker verification before they can be translated into the clinical practice.

## Graphical abstract

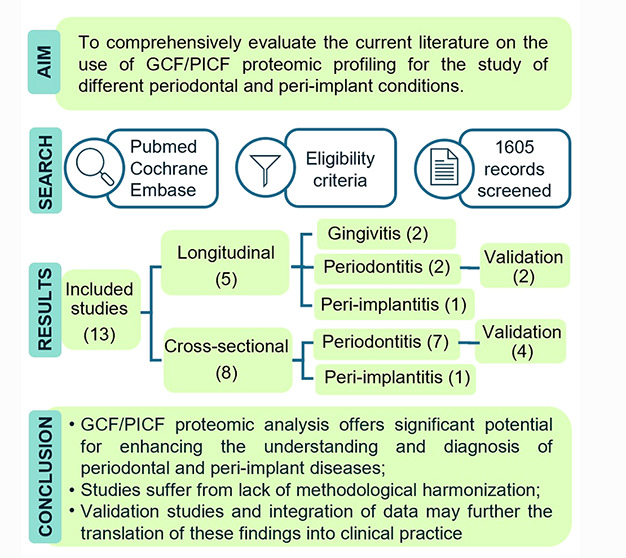

## Introduction

The diagnosis of periodontal and peri-implant diseases is based on clinical parameters, such as pocket depth (PD), bleeding on probing (BoP), suppuration, and radiographic marginal bone level (MBL).^1,2^ These parameters, however, can only assess the presence of disease once tissue destruction has already taken place. The search for new molecular biomarkers for identifying preclinical signs of the disease, therefore, has received considerable attention from the scientific community.^3–11^

Gingival crevicular fluid (GCF) and peri-implant crevicular fluid (PICF) are rich in biological markers that reflect the immune-inflammatory response to biofilm accumulation and its effects on surrounding connective tissues. Because these fluids can be collected easily and noninvasively, they have been widely proposed as promising diagnostic sources for detecting biomarkers of periodontal and peri-implant diseases.^3,5,6,9,10,12^ Inflammatory biomarkers found in GCF/PICF may offer real-time insights into the current state of the periodontal and peri-implant tissues, including inflammation, tissue remodeling, and bone metabolism.^12^ Previous investigations have explored the potential of GCF/PICF biomarkers in the detection of periodontal/peri-implant diseases, especially protein biomarkers such as matrix metalloproteinases (MMPs) and interleukins (IL), which have been related to inflammatory and bone turnover responses.^3,7,10,13–23^ Studies, however, have typically focused on the detection of individual biomarkers using antibody-based assays, which provide a limited view by analyzing only one or a few proteins at a time.^3,18^

In contrast, the use of quantitative proteomics, particularly mass spectrometry (MS), enables the detection of full sets of proteins.^6,12,24^ Proteomics is not only able to capture real-time functional states that are both genetically and environmentally shaped, it also demonstrates superior metabolic profile stability, making it distinctively informative. Proteins integrate genomic instructions and translate them into biologically actionable signals, including critical post-translational modifications, such as phosphorylation and glycosylation, which play central roles in disease mechanisms and cellular regulation.^25^ This broader and more comprehensive approach is especially valuable because periodontal and peri-implant diseases are multifactorial and influenced by complex microbial communities and host responses.^1,2^ Evaluating multiple proteins provides a more complete and accurate understanding of disease activity and progression than focusing on a single target. Carrying out this strategy allows to overcome the present limitations of disease diagnosis, currently based on signs and symptoms, toward the development of predictive, chair-side tests such as point-of-care (POC) biosensors capable of identifying disease before clinical symptoms arise.^26^ With the increasing number of studies focusing on the relationship between GCF/PICF biomarkers and periodontal/peri-implant conditions, further clarification on the present state of MS-based crevicular fluid proteomics for the study of different periodontal and peri-implant conditions is still required.

Thus, the primary aim of the present systematic review was to comprehensively evaluate the current literature on the use of GCF/PICF proteomic profiling for the study of different periodontal and peri-implant conditions. The secondary objective was to summarize methodological differences among studies in order to provide a comprehensive overview for future research in the area.

## Materials and Methods

This study has been conducted and reported in accordance with the Preferred Reporting Items for Systematic Reviews and Meta-Analyses (PRISMA) statement,^27^ and the protocol was registered with the International Prospective Register of Systematic Reviews (PROSPERO) under the number CRD42022322537.

### Focused Question

*“In systemically healthy adult patients with teeth and/or dental implants, what is the present state of MS-based proteomics of the crevicular fluid for the elucidation of periodontal/peri-implant disease mechanisms and biomarker development?”*

The focused question used in the design of this systematic review was formulated using the population, exposure, comparison and outcome (PECO) framework, as outlined below:

- (P) Population: Healthy adult patients with teeth and/or treated with dental implants.
- (E) Exposure: Teeth or implants clinically and radiographically diagnosed with gingivitis/mucositis or periodontitis/peri-implantitis.
- (C) Comparison: Teeth or implants clinically and radiographically diagnosed as being healthy.
- (O) Type of outcome measures: The main outcomes were the identification of the full content of proteins present in the crevicular fluid and changes in protein concentrations that are condition-specific, which may be used to elucidate the underlying molecular mechanisms involved in health and disease, and for the development of protein biomarkers as diagnostic and prognostic tools.

### Eligibility Criteria

**Inclusion criteria**:

- Clinical studies (cross-sectional, cohort, case-control, controlled clinical trials, and randomized clinical trials) on proteomic biomarkers found in GCF/PICF in different periodontal and peri-implant conditions (health and disease) and/or evaluating proteomic changes before and after periodontal/peri-implant treatment using MS-based approaches.
**Exclusion criteria**:
- Studies using gel-based proteomics, where MS was used only for identification;
- Studies investigating GCF/PICF proteomic profiling in specific medical conditions (i.e., diabetes, obesity, pregnancy); and
- Studies not published in English.

### Information Sources and Search Strategy

The search for clinical studies was conducted in multiple electronic databases [MEDLINE (Pubmed), Embase (Ovid), and The Cochrane Library], selected journals, and the gray literature between January 2023 and July 2024. The search strategy had no restrictions concerning time and language, and the Medical Subject Headings (MESH) terms and keywords used in each electronic database can be found in Supporting File 1.

### Selection Process

Based on the eligibility criteria, all titles and abstracts of retrieved articles were independently assessed by two reviewers (LPSSS and DRD) using Rayyan online software (https://rayyan.qcri.org). Rayyan was also used to identify duplicate publications. The examiners were previously trained and standardized to apply the inclusion and exclusion criteria. After the initial assessment, each reviewer read all studies independently. Disagreements were resolved through open discussions and, whenever a consensus could not be reached, the final decision was made by a third reviewer (FM).

### Data Items and Collection Process

Data from the studies included in the final selection were extracted by one of the authors (LPSSS), and independently assessed by two other reviewers (DRD and FM), using a predefined data extraction spreadsheet for the following variables:

- General information: first author, year of publication, country/region of origin;
- Population: number of subjects, mean age;
- Exposures and controls: case and control definition, case and control number;
- Preanalytical and analytical procedures: GCF/PICF collection (method, time, volume, site, and time point), preanalytical procedures (storage, elution, centrifugation, quantification, sample pooling), and analytical procedures (digestion, MS platform, data analysis); and
- Outcomes: number of proteins identified (discovery phase), differences in proteomic profiling (selection criteria, significant proteins in cases and controls, individual or cluster of proteins suggested as biomarkers), and biomarker validation (method, number of samples, cross-validation).

### Risk of Bias in the Included Studies and Quality Assessment

The methodological quality assessment of the included studies was independently conducted by two reviewers (LPSSS and FM) as part of the data extraction process using a modified version of the National Institutes of Health (NIH) Quality Assessment Tool for Observational Cohort and Cross-Sectional Studies and the QUADOMICS tool.^28^ The assessment table was developed to evaluate quality issues specific to omics-based diagnostic research and it is comprised of 15 items.^14^ While Q1-Q7 refer to requirements for observational clinical studies taken from the NIH, Q8-Q15 specifically refer to characteristics that confer quality to the evidence found in “-omics” studies. More specifically, Q9-Q12 refer to the verification/validation of potential candidate proteins, while Q13-Q15 to the statistical methods used to deal with confounding factors and overfitting. Overall, the items were given either a score (1) when the questions were answered “yes”, or score (0) when the answers were “no”. Items 3 (sampling procedures), 8 (handling of specimens and preanalytical procedures) and 13 (confounders) received stratified scores (0, 0.25, 0.5, 0.75, or 1), depending on their presence and level of clarity. Studies with a total score ≥13 were considered as having high quality, ≥10 and <13 as having moderate quality, and <10 as having low quality.

### Data Synthesis

Data were clustered according to the type of study, i.e., either cross-sectional or longitudinal, and outcome parameters. Descriptive summaries of the included studies were performed to conduct a synthesis of the main findings and determine methodological variations. Given the exploratory nature of the focused question, heterogeneity among included studies was anticipated, particularly regarding patient characteristics, disease definitions, crevicular fluid sampling protocols, proteomic workflows, and biomarker reporting strategies. Because the purpose of this review was to conduct a qualitative synthesis of the current state of MS-based proteomics rather than a quantitative effect estimation, heterogeneity was addressed through narrative comparative appraisal.

## Results

### Study Selection

The electronic search yielded 2087 titles, from which 482 were duplicates. After title and abstract analysis of the remaining 1605 entries, 18 studies were selected for full-text analysis, Five studies were excluded due to the following reasons: in three studies the separation of proteins was conducted by gel electrophoresis;^29–31^ one study compared GCF and saliva profiles,^32^ and one study included only healthy sites.^33^ Thus, 13 studies were regarded as eligible for analysis by the present systematic review.^12,34–45^ The study selection flowchart is displayed in Figure 1.

**Figure 1:**
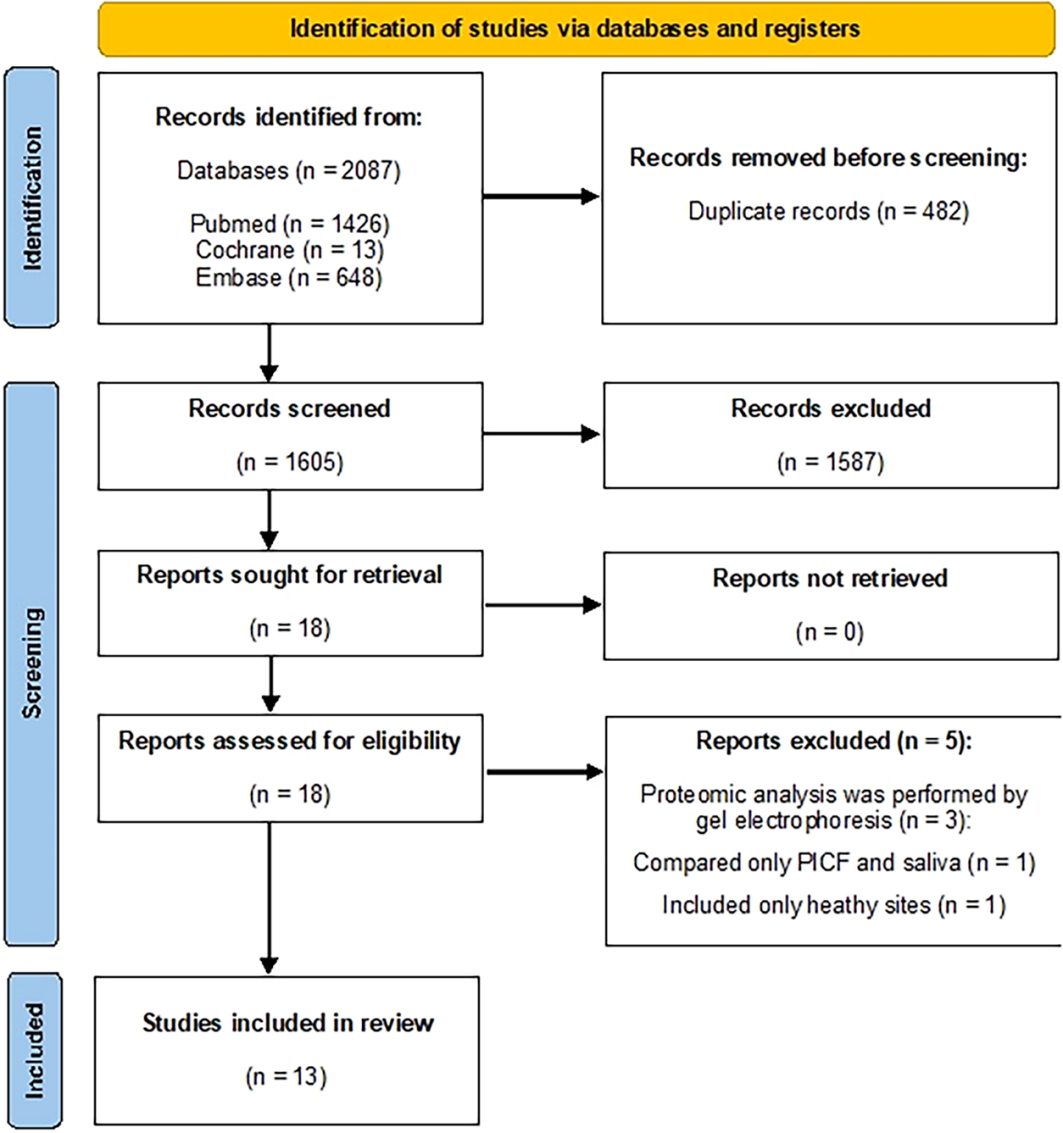
PRISMA 2020 flow diagram of the study.

### Characteristics of the Included Studies

Table 1 illustrates the general overview of included studies. Publication ranged from 2010 to 2023. Six studies were conducted in Europe, three in Asia, three in North America, and one in South America. The number of patients ranged from 10 to 190. While all studies were designed to investigate changes in the proteomic profile in the GCF and/or PICF from patients presenting different periodontal and peri-implant conditions in comparison to health, six studies were explicitly set out to identify protein biomarkers that could help to differentiate between periodontal/peri-implant health and disease,^12,34,35,40,41,43^ while three were designed to improve our understanding on the molecular mechanisms of periodontitis/peri-implantitis.^36,44,45^

**Table 1:** GFC/PICF Proteomic Profiling: Study Location, Objectives, Number of Participants and Sample Allocation Criteria^a^

authors	location	objectives	number of participants	case group (*n*; mean age)	control group (*n*; mean age)
Longitudinal Studies					
Grant et al.	Birmingham, U.K.	Describe the quantitative analysis of GCF, collected from healthy volunteers undergoing experimental gingivitis, to establish a profile of changes in proteins that may be used to compare to the inflammatory response in other subsets of the population, such as those predisposed to periodontitis.	10	GInd (*n* = 10; 21 years) 21-day experimental gingivitis (Löe, 1967)	C (*n* = 10; 21 years)
					Corresponding HT on the contralateral site
Bostanci et al.	Zurich, Switzerland	Systematically investigate the sequential protein expression in the GCF that parallels (i) the gradual conversion from pristine periodontal health to a state of established gingivitis and (ii) the resolution of gingival inflammation during reinstitution of periodontal health.	20	GInd (*n* = 10; 24.7 years)	GRes (*n* = 10; 24.4 years)
				21-day experimental gingivitis(Löe, 1967)	Debridement followed by 14 days of regular plaque control.
Guzman et al.	Texas,	Investigate candidate biomarkers for the resolution of periodontal inflammation after periodontal therapy.	10	Mod. or Adv. CP (*n* = 10; 48.8 years)	Moder. or Adv. CP (*n* = 10; 48.8 years)13 weeks postmechanical treatment
				CAL ≥ 5 mm in ≥ 30% of teeth present	
				(AAP - Armitage 1999)	
Esberg et al.	Umeå, Sweden	Determine whether PICF protein patterns could be related to covariations in implant loss, bleeding on probing, pocket depth and adjunctive EMD treatment.	25	PICF was collected before (baseline and at 3, 6, and 12 months after surgical treatment of peri-implantitis with EMD (*n* = 13; NR))	PICF was collected before (baseline and at 3, 6, and 12 months after surgical treatment of peri-implantitis without EMD (*n* = 12; NR))
Grant et al.	Birmingham,Newcastle, U.K.	Discover and validate differential protein biomarker expression in saliva and gingival crevicular fluid (GCF) to discriminate objectively between PH and plaque-induced periodontal disease states.	Birmingham = 50	Gingivitis (*n* = 10; 38 years)	Health (*n* = 10; 39 years)
			Newcastle = 140 (Total 190)	Mild to moderate CP before and after treatment (*n* = 10; 47 years)	
				Advanced CP before and after treatment (*n* = 10; 49 years)	
				Edentulous (*n* = 10; 73 years)	
Cross-sectional Studies					
Bostanci et al.	London, U.K.	Perform analysis of the GCF exudatome from healthy (PH) and periodontally diseased (AgP) sites.	10	AgP (*n* = 5; 34.6 years)	PH (*n* = 5; 32.4 years)
				Generalized aggressive periodontitis	PD < 3 mm and no radiographic evidence of alveolar bone loss.
				(AAP - Armitage 1999)	
Baliban et al.	Princeton, USA	Identify possible novel biomarkers in GCF samples from CP and PH individuals.	24	CP (*n* = 12; 56 years)	PH (*n* = 12; 49.75 years)
				(AAP - Armitage 1999)	BOP < 10% of the sites, no PD or CAL > 3 mm, and no bone loss
Baliban et al.	Princeton, USA	Identify optimal combination(s) of proteomic based biomarkers in GCF samples from CP and PH individuals and validate the predictions through known and blind test sets.	96	CP (*n* = 51; 48.02 years)	PH (*n* = 45; 45.53 years)
				(AAP - Armitage 1999)	BOP < 10% of the sites, no PD or CAL > 3 mm, and no radiographic signs of bone loss
Silva-Boghossian et al.	Rio de Janeiro, Brazil	Quantify the proteome composition of the GCF in PH and in CP sites.	10	CP (*n* = 5; 46.2 years)	PH (*n* = 5; 22.2 years)
				>10% of teeth with PD and/or CAL ≥ 5 mm and BOP.	≤10% of sites with BOP, no PD or CAL > 3 mm, although PD or CAL = 4 mm in up to 5% of the sites without BOP was allowed
				At least 5 sites with gingivitis (PD ≤ 3 mm with BOP) and 4 sites with clinical periodontal health (PD ≤ 3 mm without BOP).	
Tsuchida et al.	Tokyo, Japan	Obtain the protein profiles of GCF samples from healthy controls and patients with periodontal disease in order to identify potential biomarkers.	47	CP (31:6 mild, 7 moderate, 18 severe; 46.3 years)	PH (16; 43 years)
				(AAP - Armitage 1999)	Clinically intact gingiva
Kim et al.	Seoul, Republic of Korea	Identify periodontitis biomarkers by comparing whole GCF protein profiles between CP and PH individuals, with the aim of improving periodontal care for patients.	78	CP (*n* = 55; 50.9 years)	PH (*n* = 23; 34.7 years)
				PDs ≥ 3 mm and CAL ≥ 3 mm	Low BOP in <10% of the sites, and no sites with PD > 3 mm or CAL
Halstenbach et al.	Freiburg,Germany	Analyze and compare the proteomic composition of GCF of HT and PICF of HI and PI to gain new insights into disease biology.	14	PI (*n* = 17 samples; NR)	HI (*n* = 12 samples; NR)
				PD ≥ 6 mm, RBL ≥ 3 mm, with BOP and/or SUP	HT (*n* = 9 samples; NR)
Xiao et al.	Beijing, China	Characterize the GFC human proteome and metaproteome patterns of CP.	16	CP patients (*n* = 8)	PH patients (*n* = 8)
				(*n* = 48 samples; six per patient)	(*n* = 48 samples, six per patient)
				(AAP - Armitage 1999)	(AAP - Armitage 1999)

a GCF: Gingival crevicular fluid; PICF: Peri-implant crevicular fluid; AgP: aggressive periodontitis; BOP: bleeding on probing; C: control; CAL: clinical attachment level; CP: chronic periodontitis; EMD: enamel matrix derivative; FMBS: full mouth bleeding score; FMPS: full mouth plaque score; GI: gingival index; GInd: Gingivitis induction; GRes: gingivitis resolution; HI: healthy implants; HT: healthy teeth; PI: peri-implantitis implants; PD: probing depth; PH: periodontal health; REC: Recession; RBL: radiological bone loss; SUP: suppuration. NR: Not reported.

Five studies were longitudinal, designed to investigate changes in the proteomic profile during experimental gingivitis,^37,39^ and after periodontal^12,40^ and peri-implant treatment.^38^ One study compared healthy teeth with induced gingivitis (Gind) in the same patient using a split-mouth design,^39^ while another compared the proteomic profile during Gind and after disease resolution.^37^ The study by Guzman et al. was set out to discover candidate biomarkers for chronic periodontitis (CP) by conducting GCF proteomic profiling in sites with CP and 13 weeks after mechanical treatment.^40^ The study by Grant et al. assessed GCF collected from sites showing health and different disease states. In the case of mild to severe CP, samples were collected before and at three months after treatment to observe the alterations in protein profiles.^12^ One study evaluated protein patterns in the PICF collected before (baseline) and at 3, 6, and 12 months after the surgical treatment of peri-implantitis and observed if they could be related to covariations in implant loss, bleeding on probing, pocket depth and adjunctive enamel matrix derivative (EMD) treatment.^38^

Among the eight cross-sectional studies, seven evaluated the proteomic profile of patients with chronic periodontitis in comparison with individuals showing periodontal health (PH).^34–36,41–43,45^ One study, however, apart from collecting the GCF from sites around healthy teeth, also collected PICF from healthy implants, and implants with peri-implantitis to gain new insights into disease biology in implant rehabilitated patients.^44^

### Crevicular Fluid Collection and Preanalytical Procedures

GCF/PICF collection methods, and the preanalytical procedures conducted across the included studies are displayed in Table 2. Nine of 13 studies used Periopaper for GCF collection,^12,35–40,42^ while one study used Whatman paper strips,^38^ one reported the use of paper strips without describing which type,^45^ and two studies used absorbent paper points.^41,43^ While all studies reported a sampling time of 30 s, only six studies reported using Periotron 8000 for volume assessment,^12,36,37,42,43^ GCF/PICF collection varied broadly regarding the number and localization of sites per patient, and was conducted at different time points in the longitudinal studies.^12,37–40^ Except for Kim et al.,^41^ who did not disclose any storage information or the moment of sample elution, all the other studies reported storing samples at −80 °C before processing. Sample elution was performed before storage in six studies,^12,34,35,39,40,45^ while in six studies it was conducted after storage.^36–38,42–44^ Most studies eluted the sample in ammonium bicarbonate or phosphate buffered saline (PBS). Apart from two studies that did not report on sample centrifugation regimen,^38,44^ the centrifugation treatment varied among the remaining studies. Sample quantification was performed in nine studies using the Bradford method,^34,35,41,43,45^ bicinchoninic acid assay,^40,42,44^ or Qubit fluorometer assay,^37^ while the quantification method was not reported in the remaining four studies.^12,36,38,39^ Six studies analyzed individual samples,^34–36,38,40,43^ while in seven investigations samples were pooled together according to their clinical category and/or analytical time.^12,37,39,41,42,44,45^ Although sample digestion was conducted with Trypsin in all studies, in three studies Trypsin was combined to Lys-C.^12,41,44^

**Table 2:** GCF/PICF Collection, and Sample Preparation^a^

	Crevicular Fluid Collection					Sample Preparation					
Authors	Collection method	Collection time	Collection Volume	Collection Sites	Time point	Storage	Elution	Centrif.	Quant.	Pooling	Digestion
Longitudinal Studies											
Grant et al.	Periopaper	30 s	Periotron 8000	Maxillary 1st and 2nd premolars and 1st molar (3 sites)	At 0, 7, 14, 21, 35 days	Eluted, stored –80 °C	200 μL of 100 mM ammonium bicarbonate	Vacuum centrifuged and reduced to 200 μL	NR	Patients within a time point and test or control sites were pooled -5 test sites and 5 control sites	Trypsin
Bostanci et al.	Periopaper	30 s	Periotron 8000	Mesio and distopalatal aspects of maxillary 1st and 2nd premolars and 1st molar	GInd: At 0, 7, 14, 21 days GRes: At 21, 25, 30, 35 days	Stored –80 °C before elution	100 μL of PBS	13,000*g* for 15 min	Qubit fluorometer assay kit	- 8 pooled samples across all subjects at each time point - used for quantification. - 1 pool from all subjects and time points in each group that served as an alignment reference in the quantification analyses.	Trypsin
Guzman et al.	Periopaper, 1–2 mm subgingivally	30 s	NR	4 sites with PD > 6 and <8 mm	At baseline, 1, 5, 9, and 13 weeks after treatment	Eluted, stored –80 °C	100 μL of 100 mM ammonium bicarbonate	8161.4*g* for 10 min	Bicinchoninic acid assay	Individual samples	Trypsin
Esberg et al.	Whatman paper strips	30 s	NR	At the sulcus site with the deepest pocket	Before and at 3, 6, and 12 months postop	Stored –80 °C before elution	200 μL of sodium chloride solution	NR	NR	Individual samples	Trypsin
Grant et al.	Periopaper Stimulated saliva	30 s	Periotron 8000	Mesiobuccal aspect of maxillary canines, 1st premolars and 1st molars (6 sites)	Before and at 3 months after treatment	Eluted, stored –80 °C	400 μL of 100 mM ammonium bicarbonate	13,000*g* for 5 min	NR	Samples pooled per group	Lys-C/Trypsin
Bostanci et al.	Periopaper	30 s	Periotron 8000	AgP: 4 deepest PDs per quadrant	NR	Stored –80 °C before elution	100 μL of PBS	13,000*g* for 15 min	NR	Individual samples	Trypsin
				PH: 4 standard sites -1st molars and central incisors							
Baliban et al.	Periopaper, 1–2 mm	30 s	NR	CP: 4 sites with PD > 6 and <8 mm	NR	Eluted, stored –80 °C	100 μL of 100 mM ammonium bicarbonate	8161.4*g* for 10 min	Bradford method	Individual samples	Trypsin
				PH: 4 mesiobuccal sites of 1st molars							
Baliban et al.	Periopaper, 1–2 mm	30 s	NR	CP: 4 sites with PD > 6 and <8 mm	NR	Eluted, stored –80 °C	100 μL of 100 mM ammonium bicarbonate	8161.4*g* for 10 min	Bradford method	Individual samples	Trypsin
				PH: 4 mesiobuccal sites of first molars							
Silva-Boghossian et al.	Periopaper	30 s	Periotron 8000	CP patients: P: 5 deep sites - PD > 4 mm;	NR	Stored –80 °C before elution	150 μL of 80% ACN, 19.9% distilled water and 0.1% TFA	Sonication for 1 min	Micro Bicinchoninic acid assay	Eluted proteins from sites of the same clinical category: 3 pools for the 5 CP subjects (P, G and H sites), and one pool for the 5 PH subjects	Trypsin
				G: 5 shallow sites with BOP;							
				H: 4 shallow sites no BOP							
				PH patients: 14 buccal sites from the upper jaw							
Tsuchida et al.	Absorbent paper points	30 s	Periotron 8000	CP: 4 sites with PD > 6 and <8 mm	NR	Stored –80 °C before elution	7 M urea and 2 M thiourea including 2% CAPS and 2 mM DTT	20,000*g* for 15 min	Bradford method	Individual samples	Trypsin
				PH: Labial side of maxillary incisors without crown and restoration							
Kim et al.	Absorbent paper points	30 s	NR	Sites with periodontal pockets and inflamed gingival tissue	NR	NR	100 μL of PBS	4000*g* for 10 min at 4 °C	Bradford method	CP and PH pooled separately	Lys-C/Trypsin
Halstenbach et al.	Periopaper	30 s	NR	NR	NR	Stored –80 °C before elution	150 μL of 0.1% acid labile surfactant in 0.1 M HEPES	NR	Bicinchoninic acid assay	Samples pooled per group	Lys-C/Trypsin
Xiao et al.	Paper strips	30 s	NR	6 teeth from each patient: 4 molars and 2 incisors (6 sites per tooth)	NR	Eluted, stored –80 °C	130 μL lysis Buffer	4 °C 14,000*g* for 10 min	Bradford method	Samples pooled per tooth	Trypsin

a ACN: Acetonitrile; PBS: Phosphate-buffered saline; TFA: Trifluoroacetic acid; DTT: dithiothreitol; HEPES: N-2-hydroxyethylpiperazine-N-2-ethane sulfonic acid; GCF: gingival crevicular fluid; PICF: Peri-implant crevicular fluid; PH: Periodontal health; CP: Chronic periodontitis; P: Periodontitis; G: gingivitis; H: health; NR: Not reported.

### Proteomic Data Acquisition, Protein Selection, and Analysis

A summary of protein data acquisition, identification, characterization and quantification, as well as the methods used for protein selection can be found in Table 3. Although protein data acquisition from the GCF and PICF samples was conducted using liquid chromatography with tandem mass spectrometry (LC-MS/MS) in all studies, different combinations of LC and MS equipment were used. Only one study used the SYNAPT G2-Si high-definition mass spectrometer,^38^ while all the other studies used different models of the Orbitrap mass spectrometer. Protein/peptide labeling was conducted prior to LC-MS/MS in three studies; two studies used isobaric tags for relative and absolute quantitation (iTRAQ),^12,39^ while one used tandem mass tag (TMT).^43^ In the remaining 10 studies, LC-MS/MS was conducted without peptide labeling.

**Table 3:** Protein Data Acquisition, Identification, Characterization, and Analysis^a^

Author	Labeling	LC-MS/MS equipment	Software – Algorithm	Human database	Statistics/Bioinformatics
Longitudinal Studies					
Grant et al.	iTRAQ 8-plex label	Micro AS autosampler and Surveyor MS pump and LTQ Orbitrap XL hybrid FTMS	Proteome Discoverer sp 1.0 – SEQUEST algorithm	IPI human database v3.66	Fold changes of proteins at test sites normalized to control sites. Temporal quantitative profiles of the proteins were analyzed using PolySNAP3 software. Cluster analysis was performed by PCA.
Bostanci et al.	Label-free	Bishoff Chromatography coupled to an Eksigent nanoLC-1D and LTQ Orbitrap MS	Mascotserver 2.3 - NR	In-house database	Fold changes in abundance intensity. Cluster analysis and heat maps were generated using the R software.
Guzman et al.	Label-free	LTQ Orbitrap XL MS coupled to an Agilent 1200 Series binary HPLC pump and an Eksigent AS2 autosampler	PILOT_PROTEIN - NR	SwissPrrot	For each patient, any proteins that transitioned between presence and absence over time were considered to be candidate biomarkers. Temporal profiling was performed using a logistic function. Proteins fp (13) – fp (0) ≥ 0.5 were kept as candidate biomarkers. A MILP model was used to select the top 5 candidate biomarkers.
Esberg et al.	Label-free	nanoESI-equipped SYNAPT G2-Si high definition MS	ProteinLynx Global SERVER v.3.0.3 - NR	UniProt	Cluster analysis by the presence of proteins was performed by PCA and OPLS-DA regression. Protein functions was assessed with DAVID Bioinformatics Resource 6.8; and protein–protein interaction with STRING database.
Grant et al	iTRAQ 8-plex label	LTQ Orbitrap Velos MS	Proteome Discoverer - Mascot and SEQUEST	IPI human database	Ratio of test group to health. The quantitative profiles were analyzed using PolySNAP3. Shortlisted candidate proteins were verified by ELISA. Leave-one-out cross validation logistic regression analysis was used to determine biomarker combinations.
Cross-Sectional Studies					
Bostanci et al.	Label-free	NanoACQUITY HPLC and Q-Tof Premier MS	Protein Lynx Global Server version 2.3.5 - NR	UniProt/Swiss-Prot	The absolute quantity of each protein was expressed as fmol. Relative up-regulation or down-regulation of proteins in disease, compared to health.
Baliban et al.	Label-free	Hybrid LTQ-Orbitrap MS Coupled to an Agilent 1200 Series binary HPLC pump and an Eksigent AS2 autosampler	PILOT_PROTEIN - NR	SwissProt	Human proteins found only in at least three healthy samples or only in at least three diseased samples. Three proteins from each group were identified as biomarker candidates based on their frequent appearance in only PH or CP sample sets.
Baliban et al.	Label-free	Hybrid LQT-Orbitrap MS coupled to an Agilent 1200 Series binary HPLC pump and an Eksigent AS2 autosampler	PILOT_PROTEIN - NR	SwissProt	Number of times that a protein appears in the samples of each group and its relative frequency. A MILP optimization model was developed to identify the optimal combination of biomarkers that could clearly distinguish a blinded sample from a PH or CP patient
Silva-Boghossian et al.	Label-free	Nano-HPLC Proxeon and LTQ Orbitrap Velos MS	Proteome Discoverer 1.3 - SEQUEST	Swiss-Prot and TrEMB	Relative abundance ratio of proteins of test groups compared to PH. Proteins from PH group were considered significantly different protein level when the values observed were <0.75 for decreased abundance or >1.25 for increased abundance (*p* < 0.001).
Tsuchida et al.	TMT 6-plex Isobaric label	HPLC and LTQ-Orbitrap XL MS	Proteome Discoverer - NR	IPI human database	Severely increased periodontal disease/healthy control ratios in GCF samples (ratio >5) and slightly increased severe periodontal disease/healthy control ratios in GCF samples (ratio >2 < 5).
Kim et al.	Label-free	Q Exactive HF-X hybrid quadrupole-Orbitrap MS coupled with an Ultimate 3000 RSLC nanosystem	Proteome Discoverer 2.4 - SEQUEST-HT	UniProt	The average spectral counts of the analyses were calculated for PH and CP samples, and then the fold changes in CP compared with PH were calculated.
Halstenbach et al.	Label-free	Orbitrap MS coupled to an Easy nanoLCTM 100 UHPLC	DIA-NN (v. 1.7.12) - NR	UniProt	Only proteins found in ≥20% samples were considered. Logarithmic fold change was calculated. Only proteins with log2-changes of ±50% were considered as up- or downregulated (padj = 0.05). PLS-DA and LIMMA were applied to compare the groups.
Xiao et al.	Label-free	Orbitrap Fusion Lumos MS	Proteome Discoverer 2.4 - NR	UniProt and SwissProt	Differential proteins between control and periodontitis by DIA analysis (fold change of ≥ 1.5). PCA analysis was conducted to investigate the variation of the GCF proteome and metaproteome. Functional analysis on differential human proteins was further performed by IPA software.

a iTRAQ: Isobaric tags for relative and absolute quantitation; LTQ: Linear Ion Trap; MS: Mass spectrometer; FTMS: Fourier Transform mass spectrometer; nanoESI: nanoelectrospray ionization; TMT: tandem mass tag; HPLC: high-performance liquid chromatography; UHPLC: Ultrahigh-performance liquid chromatography PCA: Principal component analysis; GCF: gingival crevicular fluid; MILP: Mixed-integer linear programming; PH: Periodontal health; CP: Chronic periodontitis; ELISA: Enzyme-linked immunosorbent assay: IPA: Ingenuity pathway analysis; OPLS-DA: Orthogonal partial least-squares discriminant analysis; PLS-DA: Partial least-squares regression analysis; LIMMA: Linear models for microarray data; STRING: Search Tool for the Retrieval of Interacting Genes/Proteins database; DAVID: Database for Annotation, Visualization and Integrated Discovery; DIA-NN: Data-independent acquisition natural networks; NR: not reported.

Protein identification, characterization and quantification were conducted with the use of different algorithms and software packages (Proteome Discoverer, Mascotserver 2.3, PILOT_PROTEIN and Protein Lynx) against various human and bacterial databases such as the UniProt, Swissprot, TrEMB, and the International Protein Index (IPI). The selection of relevant proteins was conducted in all studies to observe differences in the proteomic profiles and, in some cases, determine possible biomarkers that could differentiate between periodontal/peri-implant conditions. Selection methods varied widely from quantitative data in fmol being analyzed in Microsoft Excel,^36^ 3-dimensional metric multidimensional scaling (MMDS) plots and dendrograms,^39^ clustering analysis and heat maps,^37^ temporal profiling,^40^ a mixed-integer linear programming (MILP) optimization model,^35^ principal component analysis (PCA) with orthogonal partial least-squares discriminant analysis (OPLS-DA) regression,^38^ Mann–Whitney U-test and Kruskal–Wallis test using the Dwass-Steel-Chritchlow Flinger method,^43^ nonparametric Kruskal–Wallis tests and Dunn’s post hoc analysis for multiple comparisons,^41^ leave-one-out logistic regression analysis to determine the receiver operating characteristic (ROC) and area under the curve (AUC) of biomarker combinations,^12^ to partial least-squares regression analysis (PLS-DA) and linear models for microarray data (LIMMA).^44^ In the present review, only the study by Esberg et al.^38^ used the Search Tool for the Retrieval of Interacting Genes/Proteins (STRING) database for protein–protein interactions while in another study the authors conducted the Ingenuity Pathway Analysis (IPA).^45^

### Summary of the Results

Table 4 presents a summary of the results, with the number of human and nonhuman proteins found, proteins selected due to their significance, candidate biomarkers, and biomarker validation procedures found in each selected study. The total number of proteins of human origin ranged from 42 to 3070. Proteins of bacterial, viral and yeast origin were also found in different amounts, ranging from 13 to 352.

**Table 4:** Outcomes, Significant Proteins, Candidate Biomarkers, Validation and Conclusions^a^

Authors	Total proteins discovered	Significant distinctive proteins	Candidate biomarkers	Validation/Verification	Conclusions
Longitudinal Studies					
Grant et al.	**16 bacterial 186 human**	The human proteins identified spanned a wide range of compartments (extracellular and intracellular) and functions. Clusters A, E and B2vi that associated with changes between the clinical parameters included neuronal and synapse associated proteins.	NR	NR	Quantitative analysis of temporal changes of proteins in GCF with a nonpresumptive approach was conducted for the first time. New proteins that highlight structural components of the gingiva were identified.
Bostanci et al.	**14 bacterial 12 fungal 7 yeast 254**	35 proteins were upregulated at day 21 versus baseline. 64 were downregulated at day 35 versus day 21. Proteins involved in cytoskeletal rearrangements, immune response, antimicrobial function, protein degradation, and DNA binding.	NR	NR	LC–MS/MS label-free quantitative proteomics is a valuable tool in the search of biologically relevant proteins potentially modulated during experimental gingivitis, and can provide better understanding of the molecular mechanisms in the induction and resolution of gingival inflammation.
Guzman et al.	**55–204** Along the experiment	A small subset of proteins was extracted, including azurocidin, lysozyme C and myosin-9 as candidate biomarkers prominent at baseline and α-smooth muscle actin as prominent 13 weeks after treatment.	**PH:** α-sm actin | γ-sm actin	Cross-validation studies yielded average predictive accuracy of 0.900 and AUC of 0.930.	High-throughput proteomic analysis can contribute to identifying end points of periodontal therapy. The candidate proteins have biological support in the literature for their importance in CP progression and recovery. Targeted studies may elucidate the mechanisms of periodontitis and the evaluation of their clinical efficacy.
			**CP:** Azurocidin, Myosin-9, Lysozyme C		
Esberg et al.	NR	52 proteins identified to differ between clusters 2 (implant loss) and 3 (success), all of them showing increased prevalence in cluster 2 compared with cluster 3.	NR	NR	The presence of two different PICF proteomic expression profiles could be demonstrated within the peri-implantitis pocket: one response associated with implant loss and BoP (cluster 2), the other associated with implant survival and EMD treatment (cluster 3).
Grant et al	**GFC:** 264 human 6 bacterial	15 candidate proteins	Metalloproteinase 9 (MMP9), α 1 acid glycoprotein (A1AGP) pyruvate kinase (PK) with the possible addition of S100A8.	ELISA Leave-one-out cross validation logistic regression analysis	The combination of four proteins, with and without age as a further parameter, can distinguish between periodontal health and disease.
	**Saliva:** 307 human 7 bacterial				
	GCF/Saliva: 95 proteins				
Cross-Sectional Studies					
Bostanci et al.	**8 viral 14 yeast 27 bacterial 101 human**	Host defense-related proteins, such as Cystatin-B and defensins were present only in health. Among the newly identified GCF proteins were L-plastin detected only in disease (15.6–12.1 fmol) and Annexin-1 detected in levels 5-fold higher in health.	Neutrophil defensin (HNP)	Neutrophil defensin (HNP) levels measured in the same samples with an ELISA kit	LC/MS technology may facilitate the characterization of GCF proteome in PH and CP, with prognostic and diagnostic value.
Baliban et al.	**30 bacterial 432 human** 123 CP only 79 PH only	11 proteins associated with PH	**PH:** Angiotensinogen, Thymidine phosphorylase, and Clusterin.	NR	LC MS/MS for large-scale comprehensive proteomic analysis may result in the identification of novel biomarkers for PH or CP.
		32 proteins associated with CP	**CP:** Neutrophil defensin-1, carbonic anhydrase-1, elongation factor-1 γ.		
Baliban et al.	**42 to 190** for each sample	Four models involving different combinations of 7 human and 3 bacterial proteins.	**PH:** Glyceraldehyde 3 phosphate dehydrogenase, thymidine phosphorylase Ig kappa chain V–I region AG	Cross-validation of the MILP model on a training set of 55 samples with an accuracy >99% to discriminate PH or CP samples	The proposed large-scale proteomic analysis and MILP model led to the identification of novel combinations of biomarkers for consistent diagnosis of periodontal status with greater than 95% predictive accuracy.
			**CP:** lysozyme C, hemoglobin subunit delta and peptidyl-prolyl cis–trans isomerase A.		
Silva-Boghossian et al.	**230** (145 in PH, 214 in P, 154 in G, 133 in H)	35 proteins showed significantly high abundance, while 4 proteins showed significantly low abundance in CP sites compared to the PH.	NR	NR	There are marked differences in the GCF proteome according to disease profile. Comprehension of the role of the identified proteins in the etiopathogenesis of periodontal disease may lead to biomarker definition.
Tsuchida et al.	**619** Unique peptide sequences	39 proteins ↑CP: (CP/PH ratio >5) 19 proteins ↑CP: (CP/PH ratio >2 < 5)	Matrix metalloproteinase 9* (MMP9), Neutrophil gelatinase-associated lipocalin (LCN2).	Western blot ELISA	Two potential novel biomarkers for periodontal disease, both of which were up-regulated proteins were identified as potential biomarkers of periodontal disease. GCF from severe periodontal disease patients exhibited higher expression of LCN2 and MMP-9 than healthy controls. ELISA also confirmed significantly higher levels of LCN2 in patients with periodontal disease than in healthy subjects.
Kim et al.	**1295** 104 CP only 4 P only	**CP:** 228 increased >5-fold 138 decreased >2-foldGalectin-10 (Gal-10) was one of the most ↑ proteins in CP patients.	Galectin-10 (Gal10)	Western blot with a Gal10 specific antibody ELISA	Gal-10 expression was increased in the GCF of patients with periodontitis and contributed to the process of osteoclastogenesis. Therefore, Gal-10 may be a candidate biomarker for periodontitis.
Halstenbach et al.	**334** bacterial	T vs I: no differentially expressed proteins.	NR	NR	Peri-implantitis sites exhibit an inflammatory fingerprint when compared to healthy implants and teeth. Healthy implants and teeth share strong similarities in their proteome. The method may pave the way to a more profound understanding of disease biology, new diagnostic and prognostic tools and patient-specific therapy.
	**2332** human	P* vs I: 59 ↑ and 31 proteins ↓ in P*.			
Xiao et al	**3070** human	368 proteins were increased in periodontitis	NR	NR	The functional activity of the GCF host proteins and microbiota was characterized and new host–microbiome signatures identified, including the alteration of butyrate metabolism, which may destroy intercellular junctions and trigger cell death of gingival epithelial cells. Valuable information at the protein level for aiding in understanding the possible molecular mechanism of periodontitis is provided.
	**752** bacterial	202 were decreased			

a NR: Not reported; GCF: gingival crevicular fluid; MILP: Mixed-integer linear programming; PH: Periodontal health; CP: Chronic periodontitis; ↑: Upregulated; ↓: Down regulated; AUC: area under the ROC curve; PICF: Peri-implant crevicular fluid; EMD: enamel matrix derivative; ↑: Upregulated; ↓: Downregulated; ELISA: Enzyme-linked immunosorbent assay; P: Periodontitis; G: gingivitis; H: health; T: health teeth; I: Healthy implants; P*: implants with peri-implantitis.

All studies identified proteins or clusters of proteins that were more expressed in health or periodontal/peri-implant samples. Six of them aimed only to describe the differences between the groups, without suggesting any biomarkers. Grant et al.^39^ identified three clusters associated with changes to the clinical parameters. Bostanci et al.^37^ found 35 proteins that were upregulated at the end of gingivitis induction phase (day 21) compared with baseline, and 64 proteins downregulated at the end of gingivitis resolution phase compared with day 21. Esberg et al.^38^ identified two major clusters with 52 different proteins showing increased prevalence in cluster *2 (associated with implant loss and BoP) when compared with cluster *3 (associated with implant survival and EMD treatment). Silva-Boghossian et al.^42^ found thirty-five proteins with significantly high abundance and 4 proteins showing significantly low abundance in CP compared to the PH sites. Halstenbach et al.^44^ found no differentially expressed proteins when healthy teeth were compared to health implants but found that 59 proteins were upregulated and 31 downregulated when sites with peri-implantitis were compared to healthy implants. Finally, Xiao et al.^45^ found 368 proteins that were increased in periodontitis while 202 were decreased.

In seven studies the authors went one step ahead as to suggest either individual proteins or group of proteins as potential biomarkers. Guzman et al.^40^ extracted a small subset of proteins as candidate biomarkers prominent at baseline and some prominent 13 weeks after treatment. Four proteins displayed strong temporal profiles, one in PH (α-sm actin | γ-sm actin) and three in CP (Azurocidin, Myosin-9, Lysozyme C). Bostanci et al.^36^ found host defense-related proteins, such as Cystatin-B and defensins, present only in health. Among the newly identified GCF proteins, L-plastin was detected only in disease while Annexin-1 was detected in levels 5-fold higher in health. The authors considered Neutrophil defensin (HNP) as putative biomarker. Baliban et al.^34^ found 11 proteins associated with PH and 32 proteins associated with CP, most of them not previously evaluated as biomarkers of periodontal conditions. The authors suggested that 3 human and 3 bacterial proteins in PH (Angiotensinogen, Thymidine phosphorylase, Clusterin, 33 kDa chaperonin, iron uptake protein A2, phosphoenolpyruvate carboxylase) and 3 human and 3 bacterial proteins in CP (neutrophil defensin-1, carbonic anhydrase-1, elongation factor-1 γ, ribulose biphosphate, carboxylase, a probable succinyl-CoA:3-ketoacid-coenzyme A transferase, or DNA-directed RNA polymerase subunit β) could be used to identify different periodontal status. Baliban et al.^35^ proposed four different solutions (models) involving different combinations of 7 human proteins and 3 bacterial proteins that were shown to be capable of discriminating between PH (Glyceraldehyde 3 phosphate dehydrogenase, thymidine phosphorylase Ig kappa chain V–I region AG) and CP samples (lysozyme C, hemoglobin subunit delta and peptidyl-prolyl cis–trans isomerase A). Tsuchida et al.^43^ identified the proteins matrix metalloproteinase 9 (MMP9) and neutrophil gelatinase-associated lipocalin (LCN2) as being upregulated in CP, and indicated that LCN2 may be a promising GCF biomarker for the detection of periodontal disease. Kim et al.^41^ found that Galectin-10 (Gal-10) was one of the most upregulated proteins in CP patients, and suggested that Gal 10 could be considered as a potential biomarker for CP in patients with periodontitis, since it contributes to the process of osteoclastogenesis by inducing molecules related to inflammation and osteoclast differentiation. Grant et al.^12^ developed panels of 3–4 analytes or 4 analytes + age to distinguish health or gingivitis from periodontitis, health from gingivitis, and mild periodontitis from advanced disease. All panels included the proteins α-1-acid glycoprotein, MMP9, S100A8, and pyruvate kinase.

The validation of individual biomarkers or cluster of biomarkers was attempted in six studies. Bostanci et al.^36^ measured neutrophil defensin human neutrophil peptide (HNP) levels in the same samples with an enzyme-linked immunosorbent assay (ELISA) kit. Guzman et al.^40^ conducted cross-validation studies that yielded an average predictive accuracy of 0.900 and an AUC of 0.930. Baliban et al.^35^ conducted cross-validation of the MILP model on a training set of 55 samples, which showed an accuracy >99% to discriminate PH from CP samples. Tsuchida et al.^43^ used Western Blotting and ELISA in the same discovery samples, while Kim et al.^41^ also used these same techniques with a Gal10 specific antibody. Grant et al.^12^ validated proteins based on the availability of ELISA kits and conducted a leave-one-out cross-validation with logistic regression analysis. In that study, while the Birmingham cohort was used for proteomics-based discovery and ELISA validation, the Newcastle cohort was used for ELISA validation only.

### Risk of Bias and Quality Assessment in Studies

Table 5 reports on the results of the quality assessment using the modified QUADOMICS tool.

**Table 5:** Quality Assessment of the Included Studies Evaluated by a Modified Version of the QUADOMICS Tool^a^ ^,^ ^14^

Item	Baliban et al.	Baliban et al.	Bostanci et al.	Bostanci et al.	Esberg et al	Grant et al.	Grant et al.	Guzman et al.	Kim et al.	Silva-Boghossian et al.	Tsuchida et al.	Halstenbach et al	Xiao et al.
1. Was the research question or objective in this paper clearly stated and appropriate?	Yes	Yes	Yes	Yes	Yes	Yes	Yes	Yes	Yes	Yes	Yes	Yes	Yes
2. Was the study population clearly specified and defined?	Yes	Yes	Yes	Yes	Yes	Yes	Yes	Yes	Yes	Yes	Yes	Yes	Yes
3. Were procedures and timing of biological sample collection with respect to clinical factors described with enough detail?	0.75	0.75	1	1.0	0.75	1	1	0.75	0.75	1	1	0.5	0.75
4. Did the authors include a sample size justification?	No	No	No	No	No	No	No	No	Yes	No	No	No	No
5. Were controls selected or recruited from the same population that gave rise to the cases (including time frame)?	Yes	Yes	Yes	Yes	Yes	Yes	Yes	Yes	Yes	Yes	Yes	Yes	Yes
6. Were the definitions, inclusion and exclusion criteria, used to identify or select cases and controls valid, reliable, and implemented consistently across all study participants?	Yes	Yes	Yes	Yes	Yes	Yes	Yes	Yes	Yes	Yes	Yes	Yes	Yes
7. Were the cases clearly defined and differentiated from controls?	Yes	Yes	Yes	Yes	Yes	Yes	Yes	Yes	Yes	Yes	Yes	Yes	Yes
8. Was handling of specimens and preanalytical procedures reported in sufficient detail and similar for the whole sample?	1	1	0.75	1	0.5	0.75	0.75	1	0.75	1	1	0.75	1
9. Is the time period between the reference standard and the index test short enough to reasonably guarantee that the target condition did not change between the two tests?	No	Yes	Yes	No	No	No	Yes	Yes	Yes	No	Yes	No	No
10. Is the reference standard likely to correctly classify the target condition?	No	Yes	Yes	No	No	No	Yes	Yes	Yes	No	Yes	No	No
11. Did the whole sample or a random selection of the sample receive verification using a reference standard of diagnosis?	No	Yes	Yes	No	No	No	Yes	Yes	Yes	No	Yes	No	No
12. Were the index test results interpreted without knowledge of the results of the reference standard?	No	Yes	No	No	No	No	Yes	No	No	No	No	No	No
13. Were key potential confounding variables measured and adjusted statistically in the analyses?	0	0	0	0	0	0	0	0	0	0	0	0	0
14. Were uninterpretable/intermediate test results reported?	Yes	Yes	Yes	Yes	Yes	Yes	Yes	Yes	Yes	Yes	Yes	Yes	Yes
15. Is it likely that the presence of overfitting was avoided?	No	Yes	No	No	No	No	Yes	Yes	No	No	No	No	No
**Total score**	**7.75**	**12.75**	**10.75**	**8**	**6.75**	**7.75**	**12.75**	**11.75**	**11.50**	**8**	**11**	**7.25**	**7.75**
**Quality evaluation**	**Low**	**Mod**	**Mod**	**Low**	**Low**	**Low**	**Mod**	**Mod**	**Mod**	**Low**	**Mod**	**Low**	**Low**

a Mod: moderate Scores: Yes (1); No (0)/13 – 15 = High quality; 10–13 = Moderate quality; 0 – 10 = Low quality.

While the totality of the studies clearly stated their objectives (Q1), and adopted replicable criteria for the selection of control (periodontal/peri-implant health) and test subjects (periodontal/peri-implant disease) (Q2), several variations were observed in the report of sample collection procedures and timing (Q3).^35,43^ Only one of the studies included a sample size justification (Q4),^41^ indicating that the majority of studies opted for samples of convenience. Although all the studies provided a clear, and consistent report on patient selection (Q5-Q7), several variations in the report of sample handling and preanalytical procedures were observed among the studies (Q8). The lack of verification/validation procedures resulted in several studies receiving a “No” score (Q9-Q12), while key confounding variables being measured and adjusted statistically in the analyses was not reported by any of the studies (Q13). Only three studies indicated that overfitting was avoided by performing the verification in a test sample different from the training sample (Q15).^12,35,40^ According to the criteria adopted, none of the studies achieved a high-quality score, six studies were considered as having moderate quality,^12,35,36,40,41,43^ and seven studies as having low quality.^34,37–39,42,44,45^

## Discussion

This systematic review is the first to comprehensively evaluate the current state of biomarker development in the fields of periodontics and peri-implantology based on GCF/PICF as the biological substrate and the use of MS-based proteomics. The findings indicate that important initial steps have been taken toward identifying molecular biomarkers and mechanisms for the diagnosis and prognosis of periodontal/peri-implant diseases. However, their integration into clinical practice will require further methodological standardization and robust validation practices.

One of the aspects that stands out from this review is the fact that only two of 13 studies dealing with crevicular fluid MS-based proteomic profiling have focused on peri-implant conditions.^38,44^ Although the complex biological structure of the periodontium that surround teeth cannot be directly compared to a site with an implant anchored directly on the alveolar bone, peri-implant diseases (mucositis and peri-implantitis) have been closely related to periodontal diseases (gingivitis and periodontitis) and treated based on similar concepts.^46^ This seems to be one of the reasons behind the limited research on implant-related conditions, since there might be the perception that the findings from the periodontal proteome might be directly translated into the peri-implant proteome. Another reason might concern PICF collection procedures, which can be impaired by prosthesis design and implant positioning and localization, demanding the establishment of specifically designed protocols to ensure enough amounts of PICF for LC-MS analyses. It is also known that peri-implant diseases are highly prevalent among implant rehabilitated individuals,^47–49^ which could impose a challenge in terms of obtaining adequate cohorts of individuals with peri-implant health. Nonetheless, future studies on PICF proteomic profiling are highly warranted to narrow the gap presently existing in our understanding of peri-implant diseases and how to treat them, and to reduce our reliance on what is already known about periodontal diseases. Studies specifically designed to investigate the peri-implant proteome may not only shed more light on the unique mechanisms of peri-implant diseases, but also provide an opportunity to establish a clearer distinction between peri-implant and periodontal diseases, reduce variability in diagnostic criteria and lead to differential treatment.

In general, the large number of human, as well as bacterial, viral and yeast proteins discovered in GCF/PICF samples is a clear indication of the effectiveness of MS-based proteomics approach. As a result, proteomic profiling and biomarker development using GCF/PICF have been showing some encouraging signs of evolution in recent years; the studies conducted by Baliban et al.^34,35^ being a good example. The analysis conducted by the authors in 2012 involved a small sample of unmatched participants and potential biomarkers selected according to their frequency discovery.^34^ Conversely, in their subsequent study of 2013, a larger sample of matched participants was used to reduce risk of bias, and a MILP model was proposed to identify optimal combinations of protein biomarkers, which were then verified/validated using independent samples through blinded and nonblinded testing.^35^ Guzman et al.^40^ benefited from this previous experience by conducting biomarker selection with the same MILP model together with temporal profiling, followed by biomarker cross-validation studies using predictive accuracy and AUC. Despite this obvious progress, findings could not be directly translated into the clinical practice due to the lack of assessments of their clinical efficacy.

Some key proteins have been considered as potential biomarkers in more than one study. Lysozyme C, cited by two studies,^35,40^ is an antimicrobial protein involved in the innate immune response that exerts bactericidal activity by hydrolyzing bacterial cell walls. It is abundant in several biological secretions, including tears, saliva, human milk, and mucus, and exhibits antimicrobial activity against both Gram-positive and Gram-negative bacteria. Previous studies have associated Lysozyme activity in crevicular fluid and saliva with periodontal diseases.^50,51^ Neutrophil defensins were also reported twice,^34,36^ which are antimicrobial peptides that are part of the innate immune response and are mainly released by activated neutrophils. They exhibit broad-spectrum antimicrobial activity against Gram-positive and Gram-negative bacteria, fungi, and some viruses by disrupting microbial cell membranes, and also contribute to the modulation of inflammatory and immune responses.^52^ Several studies have evaluated the relationship between neutrophil defensins and periodontal disease, with divergent results. Some studies have found elevated levels of defensins in the disease,^53,54^ while in others they were found to be more abundant in healthy sites.^55,56^ Baliban et al.^34,35^ suggested Thymidine phosphorylase as a potential biomarker for periodontal health. This protein may have a role in maintaining the integrity of the blood vessels. They have been shown to promote endothelial cells growth and angiogenic activity in vivo, and chemotactic activity on endothelial cells in vitro.^57^ This protein has been frequently associated with cancer.^58^ Finally, Matrix metalloproteinase-9 (MMP-9) was reported by Grant et al.^12^ and Tsuchida et al.^41^ MMP-9 is a proteolytic enzyme involved in extracellular matrix degradation and tissue remodeling. It degrades several matrix components, including collagen and gelatin, and plays an important role in inflammatory cell migration, wound healing, and angiogenesis.^59,60^ Increased expression of MMP-9 in saliva has been associated with tissue destruction in periodontal disease.^61,62^

Despite these findings, no study presented a complete workflow for biomarker development, which may explain the absence of high-level scores in the omics-based quality analysis (Table 5). The transition from the discovery phase to validation was conducted through arbitrary choices of potential biomarker candidates. Researchers tend to relate newly discovered proteins/protein networks to previous knowledge coming from studies that have used immunological and/or antibody-based methods. There is a perception that the inflammatory system of the human body tends to respond to different diseases by using the same well-known mechanisms. However, when what is already known may not be sufficient to account for all situations, when the amount of data produced is massive, and especially when new horizons are being sought, a certain level of detachment from the “old ways” is required. Thus, the current dependence on antibodies for biomarker validation biases scientific investigation toward already heavily examined targets. Similar shortcomings have been highlighted in applied medicine studies.^63^ Changing from MS technologies at the discovery stages to antibody-based approaches during verification/validation only introduces uncertainty as to whether possible failures in the process are due to technical limitations or study design. This is a critical point, as proteins isoforms and homologues may not be properly quantified, and samples with higher volumes may be required.^63^ Therefore, conventional pipelines for proteomics-based biomarker development should involve not only the use of independent samples/populations, but also the combination of untargeted and targeted LC-MS-based technologies.^63^

MS technologies are currently the most effective ways to identify the full-protein contents in biological samples. However, it is not impossible that some proteins may not have been identified in the samples studied due to technological issues or bias introduced during the preanalytical phase, both keys to the process. The preanalytical processing of samples can have a significant impact on the measurable molecular output. In the present analysis, although GCF/PICF collection and the preanalytical procedures conducted in selected studies followed similar and consistent protocols, important differences were still present. While it is difficult to discern the effect that these differential approaches might have on the development of biomarkers, it seems clear, however, that higher levels of standardization will be required to prevent technical heterogeneity emerging due to different experiment times, handlers, reagents, and instruments.^64,65^ For instance, unreliable sample processing methods could affect the identification of low abundance proteins, which are fundamental for the detection of initial disease processes.^25^ Besides, quantitative proteomics can be performed either with label-free or label-based technologies prior to LC-MS/MS analyses. Label-based methods use stable isotopic labels to tag proteins or peptides prior to LC-MS/MS analyses to enhance the sensitivity and multiplexing capabilities of protein discovery. However, the additional steps required for labeling increase sample preparation complexity, significantly increasing costs, especially when a large number of samples is used. Thus, apart from three studies,^12,39,43^ simpler and more cost-effective label-free methods were conducted by most studies. The drawback, however, is that they are potentially more prone to variability, as they are highly dependent on the quality of sample preparation and the consistency of the mass spectrometry. The MS/MS spectra were matched to theoretical spectra from in-silico digests of proteins and searched against protein databases (e.g., UniProt, SwissProt, IPI) with use of algorithms (e.g., Mascot, SEQUEST, OMSSA) within different software packages (Proteome Discoverer, Pilot Protein, data-independent acquisition neural networks (DIA-NN) and Protein Lynx). Despite the effectiveness of such methods, it would seem logical, however, to expect that the capabilities of different databases and algorithms will eventually have to be integrated in order to provide more universally accepted protein data. Only when such shortcomings can be overcome, validation using targeted MS, which allows for a much more sensitive, specific, and functional detection, may be applied.

The identification of false-positive proteins may also occur, reinforcing the importance of more comprehensively understanding the mechanisms behind the identified proteins and the pathways that are being activated/inhibited. Understanding the role of the identified proteins in the etiopathogenesis of periodontal/peri-implant diseases is also an essential part in biomarker development.^42^ Differences in molecular function seem to be of fundamental importance to shed light on the biological processes that take place. The functional analysis of the proteins reported in the included studies suggest the presence of biological mechanisms involved in inflammatory regulation, host defense, and tissue remodeling pathways, supporting their potential biological relevance in disease progression. The massive amount of quantitative protein data within given conditions will also require advanced data analysis supported artificial intelligence (AI)-enhanced platforms to generate information regarding protein–protein interactions (PPIs) to further our understanding of their functional meaning, defense and pathogenicity mechanisms, as well as therapeutic targets.^66^

When interpreting the results of this systematic review, some limitations need to be considered. Although the search of the literature for the identification of studies that could contribute to answering the focus question was conducted as thoroughly as possible, there is always the possibility that some studies may have remained outside the scope of this review. Furthermore, considerable heterogeneity across the included studies can be observed,^65^ particularly regarding case definitions for periodontal and peri-implant diseases, variability in GCF and PICF collection procedures (including sampling site selection, collection time, and contamination control), differences in sample preparation and protein extraction workflows, as well as the use of distinct mass spectrometry strategies, bioinformatic pipelines, and protein identification criteria. Additional variability was related to differences in reported protein panels, validation strategies, and thresholds used to define differential expression. These methodological differences may partly explain inconsistencies in biomarker reporting, limit direct comparison among studies, and currently challenge reproducibility and clinical translation. In this context, a limitation of this review is that such heterogeneity precluded quantitative synthesis and restricted stronger comparisons of biomarker findings.

Considering the findings of the present review, it seems clear that future studies should endeavor to identify associations between the proteomic composition found in GCF and PICF and clinically established periodontal and peri-implant conditions, respectively. They should also address variations in the proteomic profile within the same clinical condition (e.g., within health) and within the same individual who presents different clinical conditions. To further our understanding of the dynamics of crevicular fluid proteomic profile, longitudinal monitoring of the same individual/site over time is also warranted. Furthermore, the study of relevant biological pathways identified in proteomic studies should also be explored in experimental studies aiming at developing more effective strategies for the identification, prevention, and treatment of periodontal and peri-implant diseases. To that aim, multicentric studies with large populations, with adequate representation of each periodontal and peri-implant state, are essential to increase the statistical power and robustness of comparisons, allowing for better discrimination between periodontal and peri-implant health and disease.

## Conclusions

MS-based proteomic analysis of the crevicular fluid offers significant potential for enhancing the understanding and diagnosis of periodontal and peri-implant diseases. The present findings emphasize the need for methodological harmonization, including standardized protocols for GCF/PICF collection, harmonized proteomic workflows, multicenter longitudinal validation studies, and targeted mass spectrometry approaches for biomarker verification. Moreover, integration of data from multiomics studies and different cohorts may further the translation of these findings into clinical practice and support the development of point-of-care diagnostic platforms.

## Supplementary Material



## Data Availability

The data underlying this study are available in the published article and its Supporting Information.

## Notes

Medical Subject Headings (MESH) terms and keywords used in each electronic database (PDF)

## List of Abbreviations

AI: artificial intelligence
AUC: area under the curve
BoP: bleeding on probing
CP: chronic periodontitis
DIA-NN: data-independent acquisition neural networks
DNA: deoxyribonucleic acid
ELISA: enzyme-linked immunosorbent assay
EMD: enamel matrix derivative
Gal-10: galectin-10
GCF: gingival crevicular fluid
Gind: induced gingivitis
HNP: human neutrophil peptide
ILs: interleukins
IPA: ingenuity pathway analysis
iTRAQ: isobaric tags for relative and absolute quantitation
LCN2: neutrophil gelatinase-associated lipocalin
LC-MS/MS: liquid chromatography with tandem mass spectrometry
LIMMA: linear models for microarray data
MBL: marginal bone level
MESH: medical subject headings
MILP: mixed-integer linear programming
MMDS: metric multidimensional scaling
MMPs: matrix metalloproteinases
MMP9: matrix metalloproteinase 9
MS: mass spectrometry
NIH: National Institutes of Health
OPLS-DA: orthogonal partial least square discriminant analysis
PBS: phosphate buffered saline
PCA: principal component analysis
PD: pocket depth
PECO: population, exposure, comparison, outcome
PH: periodontal health
PICF: peri-implant crevicular fluid
PLS-DA: partial least squares regression analysis
PPIs: protein–protein interactions
PRISMA: preferred reporting items for systematic reviews and meta-analyses
PROSPERO: international prospective register of systematic reviews
ROC: receiver operating characteristic
STRING: search tool for the retrieval of interacting genes/proteins
TMT: tandem mass tags
