# Supplementary material for: MS-Based Crevicular Fluid Proteomics for the Study of Periodontal and Peri-Implant Conditions: A Systematic Review
Source: J Proteome Res. 2026 Jun 24;25(7):3217–33. doi: 10.1021/acs.jproteome.5c01217 (PMC13339773; doi:10.1021/acs.jproteome.5c01217)
Supplement: Supplementary file 1 [file pr5c01217_si_001.pdf]

SUPPORTING INFORMATION

MS-based crevicular fluid proteomics for the study of periodontal and peri-implant conditions: A systematic review.

Laís Souza[1], Debora Dias[2], Giuliano Cesar[3], Debora Bianco[4], Mauricio Araujo[5], Flavia Matarazzo[6]*

[1] Department of Dentistry, State University of Maringa – CEP:87013-010 – Maringa, Brazil. Email: laisps3@gmail.com. ORCID: 0009-0008-5884-2653

[2] Department of Dentistry, State University of Maringa – CEP:87013-010 – Maringa, Brazil. Email: debora.reis.dias@gmail.com. ORCID: 0000-0002-5387-1753

[3] Department of Dentistry, State University of Maringa – CEP:87013-010 – Maringa, Brazil. Email: giu.cesar112@gmail.com ORCID: 0000-0001-7449-7601

[4] Department of Dentistry, State University of Maringa – CEP:87013-010 – Maringa, Brazil. Email: d.almeidabianco@gmail.com ORCID: 0009-0005-2053-1540

[5] Department of Dentistry, State University of Maringa – CEP:87013-010 – Maringa, Brazil. Email: odomar@hotmail.com ORCID: 0000-0003-2224-982X *7*

[6] Department of Dentistry, State University of Maringa – CEP:87013-010 – Maringa, Brazil. Email: flamatarazzo@gmail.com ORCID: 0000-0001-5342-0883

	Table of contents	Page
**Supplementary File 1**	Medical Subject Headings (MESH) terms and keywords used in each electronic database	S2

Supplementary File 1

PUBMED:

((((((((((((((human) OR (humans)) OR (homo sapiens)) OR (adults)) OR (adult)) OR (teeth)) OR (tooth)) OR (dental implants)) OR (peri-implant)) OR (periodontium)) OR (periimplant)) AND (((((((((((periodontal disease) OR (periodontal diseases)) OR (gingivitis)) OR (periodontitis)) OR (peri-implant disease)) OR (peri-implant diseases)) OR (peri-implant mucositis)) OR (peri-implantitis)) OR (periimplant disease)) OR (periimplant mucositis)) OR (periimplantitis))) AND (((((((periodontal health) OR (peri-implant health)) OR (periimplant health)) OR (health)) OR (healthy implants)) OR (healthy individuals)) OR (healthy subjects))) AND (((((((peri-implant crevicular fluid) OR (gingival crevicular fluid)) OR (crevicular fluid)) OR (fluid crevice)) OR (peri-implant sulcular fluid)) OR (periimplant crevicular fluid)) OR (periimplant sulcular fluid))) AND ((((((((((((((proteomic analysis) OR (proteome)) OR (proteomics)) OR (proteins)) OR (tandem mass spectrometry)) OR (mass spectrometry-mass spectrometry)) OR (mass spectrometry mass spectrometry)) OR (mass spectrometry, tandem)) OR (mass spectrometry)) OR (liquid chromatography-mass spectrometry)) OR (liquid chromatography)) OR (LC-MS)) OR (LC-MS/MS)))

EMBASE:

(**'human'**/exp OR **'human'** OR **'human being'** OR **'human body'** OR **'human race'** OR **'human subject'** OR **'humans'** OR **'man (homo sapiens)'** OR **'homo sapiens'** OR **'adult'**/exp OR **'tooth'**/exp OR **teeth** OR **'tooth implant'**/exp OR **'bicon'** OR **'grafton'** OR **'straumann mini'** OR **'straumann pure'** OR **'swish active'** OR **'swish tapered'** OR **'variobase'** OR **'dental implant'** OR **'dental implants'** OR **'implant, teeth'** OR **'implant, tooth'** OR **'implants, teeth'** OR **'implants, tooth'** OR **'intramucosal dental implant'** OR **'teeth implant'** OR **'teeth implants'** OR **'tooth implant'** OR **'tooth implants'** OR **'peri implant'** OR **'periodontium'**/exp OR **'parodontia'** OR **'parodontic tooth'** OR **'parodontium'** OR **'periodontal slide'** OR **'periodontal space'** OR **'periodontium'** OR **periimplant**) AND (**'periodontal disease'**/exp OR **'dental loss'** OR **'dental migration'** OR **'dental mobility'** OR **'furcation defects'** OR **'mesial movement of teeth'** OR **'paradontal disease'** OR **'paradontopathy'** OR **'paraodontopathy'** OR **'parodentopathy'** OR **'parodontal disease'** OR **'parodontium disease'** OR **'parodontive tissue disease'** OR **'peridontal disease'** OR **'peridontal tissue disease'** OR **'peridontium disease'** OR **'periodontal atrophy'** OR **'periodontal attachment loss'** OR **'periodontal disease'** OR **'periodontal diseases'** OR **'periodontal infection'** OR **'periodontium disease'** OR **'periodontopathy'** OR **'tooth loss'** OR **'tooth migration'** OR **'tooth mobility'** OR **'tooth movement'** OR **'gingivitis'**/exp OR **'acute gingivitis'** OR **'chronic gingivitis'** OR **'crevicular fluid'** OR **'fluid, gingiva crevice'** OR **'gingiva crevice fluid'** OR **'gingiva inflammation'** OR **'gingiva pocket'** OR **'gingival crevicular fluid'** OR **'gingival inflammation'** OR **'gingival pocket'** OR **'gingivitis'** OR **'gingivitis syndrome'** OR **'periodontitis'**/exp OR **'paradontitis'** OR **'parodontitis'** OR **'peridontitis'** OR **'periodontitis'** OR **'peri-implant disease'** OR **'implant complication'**/exp OR **'implant complication'** OR **'implant disease'** OR **'implant failure'** OR **'peri implant disease'** OR **'periimplant disease'** OR **'periimplantitis'**/exp OR **'dental implant inflammation'** OR **'dental implantitis'** OR **'dental peri implant**

inflammation' OR **'dental periimplant inflammation'** OR **'implantitis'** OR **'peri-implantitis'** OR **'periimplantitis'** OR **'peri-implant mucositis'**/exp OR **'peri implant mucosal inflammation'** OR **'peri implant mucosal inflammatory response'** OR **'peri implant mucositis'** OR **'peri mucositis'** OR **'peri-implant mucositis'** OR **'periimplant mucosal inflammation'** OR **'periimplant mucositis'** OR **'perimucositis'**) AND (**'periodontal health'**/exp OR **'peri-implant health'** OR **'health'**/exp OR **'health'** OR **'oral health'** OR **'physical health'** OR **'somatic health'** OR **'normal human'**/exp OR **'healthy adult'** OR **'healthy human'** OR **'healthy humans'** OR **'healthy patient'** OR **'healthy people'** OR **'healthy person'** OR **'healthy subjects'** OR **'healthy volunteer'** OR **'healthy volunteers'** OR **'human, normal'** OR **'normal human'** OR **'normal humans'** OR **'normal subject'** OR **'normal subjects'** OR **'normal volunteer'** OR **'normal volunteers'** OR **'healthy subject'** OR **'healthy implant'**) AND (**'peri-implant crevicular fluid'** OR **'gingival crevicular fluid'** OR **'crevicular fluid'** OR **'fluid crevice'** OR **'peri-implant sulcular fluid'** OR **'periimplant crevicular fluid'**) AND (**'proteomic analysis'** OR **'proteome'**/exp OR **'proteome'** OR **'proteomics'**/exp OR **'prote-omics'** OR **'protein omics'** OR **'proteinomics'** OR **'proteomics'** OR **'protein'**/exp OR **'alpha protein'** OR **'cationic protein'** OR **'delta protein'** OR **'endogenous protein'** OR **'kationic protein'** OR **'protein'** OR **'protein accumulation'** OR **'protein particle'** OR **'proteins'** OR **'soluble protein'** OR **'specific protein'** OR **'total protein'** OR **'tandem mass spectrometry'**/exp OR **'tandem mass spectrometry'** OR **'mass spectrometry-mass spectrometry'** OR **'mass spectrometry mass spectrometry'** OR **'mass spectrometry, tandem'** OR **'mass spectrometry'**/exp OR **'liquid chromatography'**/exp OR **'liquid chromatography-mass spectrometry'**/exp OR **'hplc-ms'** OR **'lc-ms'** OR **'lc-ms/ms'** OR **'uplc-ms'** OR **'liquid chromatography-mass spectrometry'** OR **'liquid chromatography-tandem mass spectrometry'** OR **'liquid chromatography-mass spectrometer'**/exp OR **'liquid chromatography/mass spectrometry (lc/ms) analyser ivd, automated'** OR **'hplc-ms device'** OR **'lc-ms device'** OR **'lc-ms system'** OR **'liquid chromatography-mass spectrometer'** OR **'liquid chromatography-mass spectrometry system'**)

COCHRANE:

ID Search Hits
#1 “human” OR "homo sapiens" OR adult OR tooth OR teeth OR "dental implant" OR periodontium OR periimplant OR peri-implant 1157621
#2 "periodontal disease" OR gingivitis OR periodontitis OR "peri-implant disease" OR "peri-implant mucositis" OR peri-implantitis OR "periimplant disease" OR "periimplant mucositis" OR periimplantitis 13145
#3 "periodontal health" OR "peri-implant health" OR "periimplant health" OR health OR "healthy implants" OR "healthy individuals" OR "healthy subjects" 458533
#4 "peri-implant crevicular fluid" OR "gingival crevicular fluid" OR "crevicular fluid" OR "fluid crevice" OR "peri-implant sulcular fluid" OR "periimplant crevicular fluid" OR "periimplant sulcular fluid" 1357
#5 "proteomic analysis" OR proteome OR proteomics OR proteins OR "tandem mass spectrometry" OR "mass spectrometry-mass spectrometry" OR "mass spectrometry mass spectrometry" OR "mass spectrometry, tandem" OR "mass spectrometry" OR "liquid chromatography-mass spectrometry" OR "liquid chromatography" 51235
#6 #1 AND #2 AND #3 AND #4 AND #5 13
